# Exploring physiological signals on people with Duchenne muscular dystrophy for an active trunk support: a case study

**DOI:** 10.1186/s42490-019-0032-x

**Published:** 2019-12-09

**Authors:** Stergios Verros, Laura Peeters, Arjen Bergsma, Edsko E. G. Hekman, Gijsbertus J. Verkerke, Bart F. J. M. Koopman

**Affiliations:** 1 Department Biomechanical Engineering, University of Twente, Drienerlolaan 5, 7522 NB Enschede, the Netherlands; 2 Department of Rehabilitation, Radboud University Medical Center, Reiner Postlaan 4, 6500 HB Nijmegen, the Netherlands; 3 University of Groningen, University Medical Center Groningen, Hanzeplein 1, 9713 GZ Groningen, the Netherlands

**Keywords:** Duchenne muscular dystrophy, Control interface, Trunk support

## Abstract

**Background:**

Arm support devices are available to support people with Duchenne muscular dystrophy (DMD), but active trunk support devices are lacking. An active trunk support device can potentially extend the reach of the arm and stabilize the unstable trunk of people with DMD. In a previous study, we showed that healthy people were able to control an active trunk support using four different control interfaces (based on joystick, force on feet, force on sternum and surface electromyography). All four control interfaces had different advantages and disadvantages. The aim of this study was to explore which of the four inputs is detectably used by people with DMD to control an active trunk support.

**Results:**

The results were subject-dependent in both experiments. In the active experiment, the joystick was the most promising control interface. Regarding the static experiment, surface electromyography and force on feet worked for two out of the three subjects.

**Conclusions:**

To our knowledge, this is the first time that people with DMD have engaged in a control task using signals other than those related to their arm muscles. According to our findings, the control interfaces have to be customised to every DMD subject.

## Background

People with Duchene muscular dystrophy lose their muscle functions progressively [1]. It affects one out every 6000 boys [2]. The current life expectancy has increased to about 30 years of age due to, among others, the use of corticosteroids which slow down disease progression [3]. However, the Health Related Quality of Life (HRQoL) for people with DMD is lower than healthy people [4].

Passive trunk support can increase the range of motion, stabilise the trunk, and reduce trunk muscle activation [5, 6]. However, as the DMD progresses, the patients’ muscle capacities decrease and additional assistance is needed by means of an active trunk support. To operate an active device, intention detection control interfaces are needed. Several control interfaces have been used to control active assistive devices [7]. Hand joysticks are useful for people with disabilities to con- trol powered wheelchairs and external robotic arms [8, 9]. Also, force-based control and surface electromyography are promising control strategies for people with DMD [7, 10]. Thus, we developed an active trunk support experimental setup to study and compare the performance differences of four different control interfaces of healthy males [11]. The control interfaces used were the joystick (J), force on sternum (FS), force on feet (FF) and surface electromyography (E). The results showed that all those control interfaces have the potential to be used for an active trunk support. In the current study, we wanted to explore which of the four inputs can be detectably used by people with DMD to control an active trunk support.

## Results

### Active experiment

Subject one could perform the experiment with the (J) but not with the (FS) because he could not relax his muscles sufficiently in the device. This led to non- optimal gravity compensation values and consequently an inability to control the active trunk support properly by measuring (FS). Due to technical problems with the force plate, the threshold of the measured force under the feet was higher than the force that the participant could produce with his feet and therefore he was unable to control the active trunk support setup. He could perform only 1.5 blocks out of 3 in total with the (E), because the control was too exhausting.

Subject two had spinal fusion hence his physical point of rotation was misaligned with the point of rotation of the active trunk support. He performed the experiment without the active trunk support with the (J) and the (E). (FS) could not be performed because he was not seated in the trunk support and (FF) failed for the same reason as subject one.

Subject three could only perform the experiment with the (J). (FS) also failed due to unreliable gravity compensation values as we saw in subject one. (FF) failed for the same reasons as described for subjects one and two. Spikes on the (E) signals and co-contraction of the muscles were the reasons that (E) failed for subject three.

### Static experiment

Subject one could perform the experiment with (E) but not with (FF). Stabilizing the cursor on the screen (i.e., to control the movement) was difficult by pushing against the force plate with his feet, because of equinovarus feet (lateral and up- wards).

Subject two could perform the experiment with (FF) but not with (E) because there were spikes on the signal when the subject relaxed his muscles. These spikes made it difficult to stabilise the cursor on the screen due to the fact they were unintentional and the subject could not control them.

Subject three could perform the experiment with both (FF) and (E). Regarding the (FF), the subject adapted quickly and no significant adjustments were made to tune the controller. The (E) was weak from the tibialis anterior and there were spikes on the (E) signals. Consequently, the (E) sensors were moved to the left and right quadriceps muscles but at the end of the experiment, the subject experienced cramps in the quadriceps muscles.

Table 1 summarises both experiments and the performance with each control interface.

**Table 1:** Feasible control interfaces for both experiments

	Subject one	Subject two	Subject three
Active experiment	(J), (E) (only 1,5 out of three blocks)	(J), (E)	(J)
Static experiment	(E)	(FF)	(FF), (E)

## Discussion

The aim of this research was to investigate which control interface can be used by people with DMD to control an active trunk support. For that reason, we performed two tracking task experiments with three subjects with DMD.

A (J) is not ideal for controlling a trunk support device because it requires a hand to operate the (J) and thus this hand cannot be used for other functions like grasping. Hence, (E) and (FF) were investigated in the static experiment. After tweaking the experimental procedure, two out of three participants were able to control the ball on the screen in the static experiment. Also, two participants were able to perform the experiment using the (E) based control interface. Only subject one was consistent with the (E) control. Subject two could perform the active experiment with (E) (but without the device), yet he was unable to perform the static experiment successfully while using the (E) interface. Subject three could not perform the active experiment with the (E) on the gastocnemius/tibialis muscles but after placing the (E) electrodes on the quadriceps muscle in the static experiment he could perform the experiment even though at the end he experienced cramping in both quadriceps muscles. He also pointed out that he could not continue the experiment for more than one block.

**Fig. 1:**
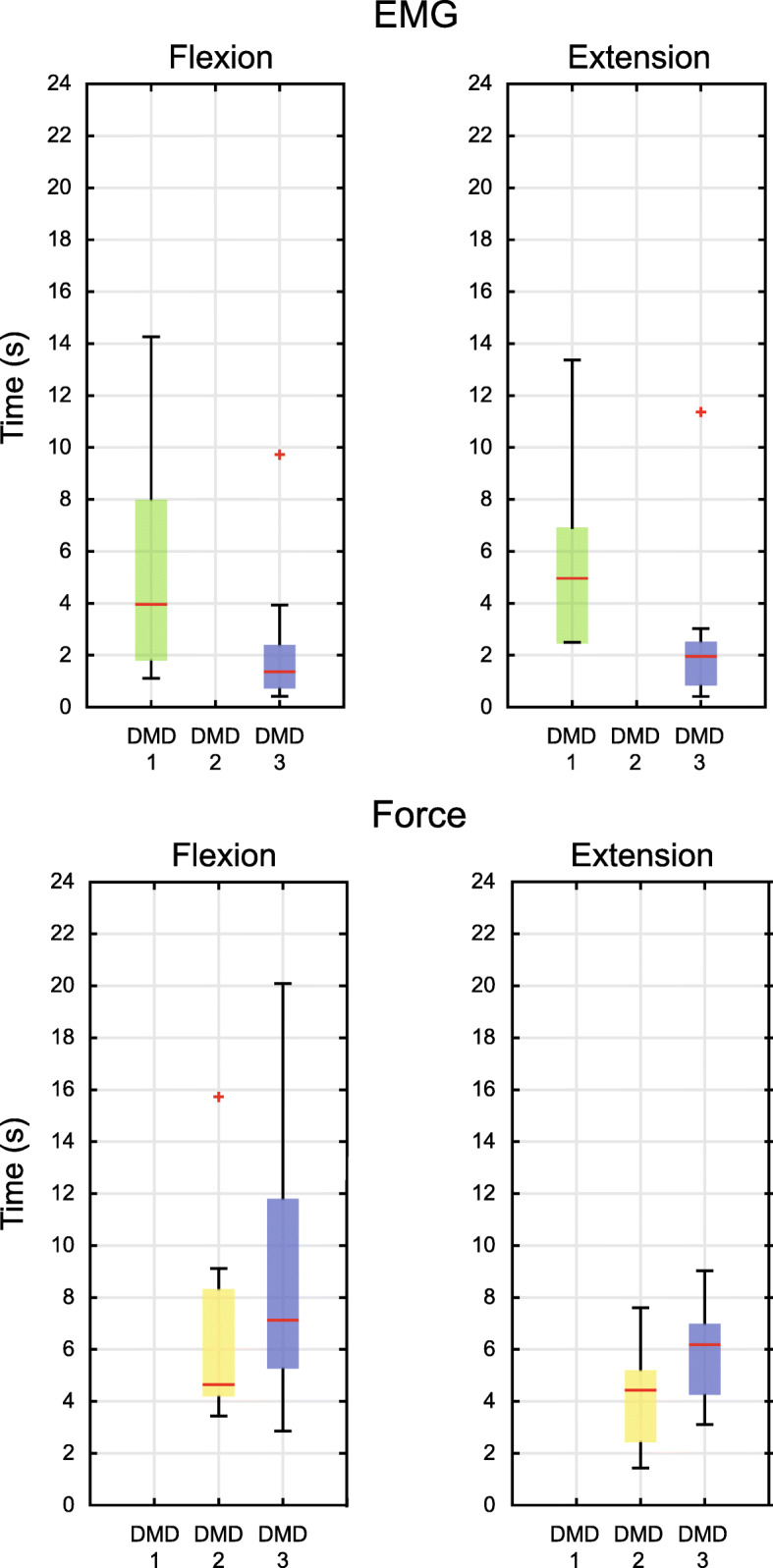
Measured Time box plots. This figure shows measured time of (E) and (FF) control for the static experiment

Previous studies have shown that force- and surface electromyography-based con- trol interfaces can be used for controlling an active elbow support on people with DMD whereby the (J) is only used for familiarisation and not for comparison [7]. Both control interfaces proved to be feasible solutions for the three adults who par- ticipated in the experiment with a 100% completion rate for forced-based control interface and 95% for surface electromyography-based control interface. In our re- search, the only control interface which worked in all cases was the (J). A major issue that we encountered was the anxiety the subjects felt when using the active trunk support. They could not support their own weight across the whole range of trunk movements due to muscle weakness and therefore the potential instability without a trunk support could have a harmful result. As a consequence, they might not have been able to relax their muscles during the gravity compensation phase. Moreover, people with DMD have significant amounts of fatty tissue concentrated on the abdominal area, which impedes them in trunk flexion. The combination of these two factors probably resulted in non-optimal gravity compensation values for the force-control interfaces and could be a reason why they could not use the (FS) control interface properly. Controlling with leg muscles might have been complicated by the fact the DMD subjects had already been wheelchair bound for more than 10 years. This means that their leg muscles had already been inactive for quite some time, with the consequence of further deterioration of the muscles due to disuse, and possible loss of motor control. Additionally, muscle fatigue sets in earlier because of muscle weakness and disuse. The opposing reasons, together with the progressive disease severity, cause the movement time of each control interface for each subject in the static experiment to be longer (Fig. 1) than that of healthy participants found in the literature, except for the (E) performed by the third subject who achieved lower movement times than the healthy controls [11]. An explanation could be the exceptional immediate familiarization of the subject with the (E) control interface. Moreover, the individual results might be a consequence of the difference in the severity of the disease among the subjects.

Time and fatigue were crucial factors in the static experiment. Tuning of the parameters was time consuming in some cases, but time to find the right parameters was limited due to rapid fatigue. Thus, the signals should be investigated further with multiple sessions of the same experiment. Also, changing to adaptive control and training might help in tuning the parameters more effectively [12]. We did not experience this in the experiment with the healthy participants where the tuning parameters were set at constant throughout the whole experiment with values based on a two subject pre-experimental procedure. Customised tuning of each control interface for people with DMD should be based on factors related to disease stage and severity.

Furthermore, the muscles of the DMD subjects’ legs are not used to contracting since they are confined to a wheelchair for many years. Their leg muscle capacity is low and fatigue occurs rapidly. Additionally, motor control can be lost with time, causing co-contraction as seen in subject three. A known saying that is applicable to people with DMD is ‘Use it or lose it’ [13]. To avoid such a phenomenon, [14] they should follow an assisted bicycling training of legs and arms. Consequently, rehabilitation should focus on keeping the muscles as active as possible, starting at the early stages of the DMD, as well as on the trunk section.

## Conclusions

Two experiments were performed with three people with DMD with the aim to investigate the use of different control interfaces with an active trunk support device. Based on the results of the active experiment, it is clear that the (J) was a feasible solution for all subjects. Alternatively, (FF) as well as (E) can also be used as control interfaces, but both of them need customized tuning for each subject.

## Methods

### Test set-up

We performed two experiments with three people with DMD. The first one was an active feasibility study. In this experiment we used the same protocol as in the experiment with healthy people [11]. While healthy people were able to control the active trunk support setup, the participants with DMD were unable to perform the same movements. Therefore we took a step back and performed a second, static experiment with the aim to give more weight to the optimisation of the control interfaces without the active trunk support setup. In this static experiment, we chose to optimise two control interfaces from the active study namely, (FF) and (E). The (J) performed well, the (FS) performed unsatisfactory, so both needed no further testing. To optimize the performance of (FF) and (E) a static experiment was performed. Additional advantage of the focus on these two control interfaces was a limitation of the exposure of DMD-patients.

### Participants

Three people with DMD, between 21-30 years of age, participated in the two exper- iments. Informed consent was given by all the participants prior to participation. The medical Ethics Committee of Radboud University Medical Center approved the study and the protocol design (NL53143.091.15). Table 2 summarises the sub- jects’ characteristics. Subjects were recruited through the outpatient clinic of the Radboud University Medical Center Nijmegen. Participants were included if they were older than 12 years of age, non-ambulant, able to understand instructions and if they had a genetically confirmed diagnosis of DMD. Subjects with other dis- abling diseases affecting the trunk, head or arms, needing continuous ventilation and participating in another study at the time, were excluded.

**Table 2:** Physical characteristics of the subjects

	Subject one	Subject two	Subject three
Age [years]	21	21	30
Age of diagnosis [years]	2	2	2
Age wheelchair confined [years]	10	11	17
Scoliosis	Mild	Mild	No
Surgical scoliosis correction (age [years])	No	Yes (17)	No
Corticosteroid use	Yes	Yes	No
Brooke scale	5	5	5

### Experimental design

#### Active and static experiment

**Fig. 2:**
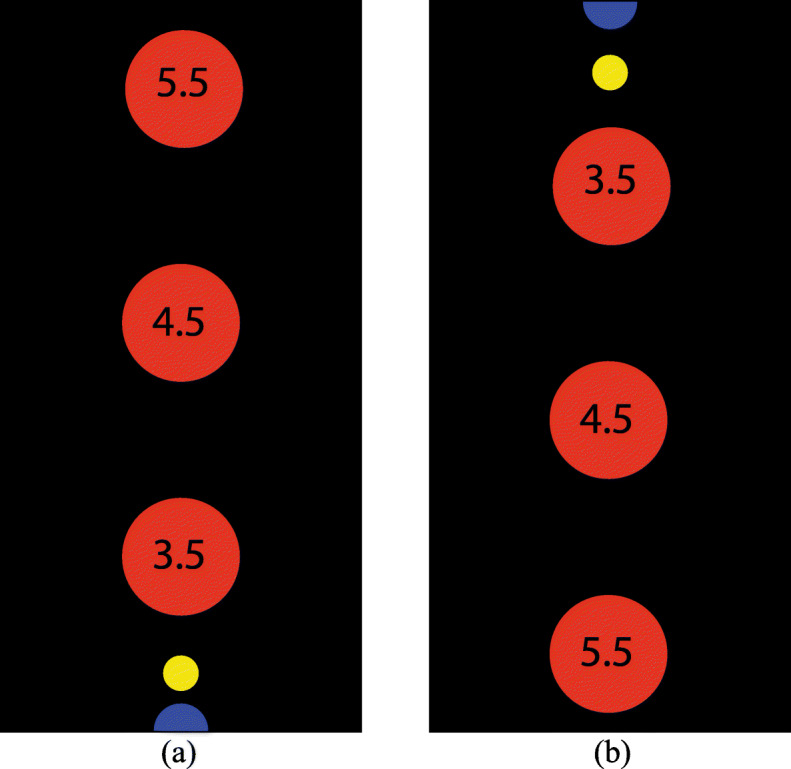
Graphical user interface. This figure shows the representation of the Graphical User Interface with target position (red circle) at different distances, the home position (blue circle) and the cursor (yellow circle). (**a**) for flexion (**b**) for extension

The experimental design was similar to the previous study [11]. A visual feedback was introduced to the subjects through a screen. Initially, the cursor (Fig. 2, yellow circle) was at the home position 1 (Fig. 2, blue circle). When the word “GO” was displayed on the screen it indicated the start of a trial. At a random time between one to three seconds, a target (Fig. 2, red circle) appeared randomly at one of the three locations on the screen. The subjects had to steer the cursor into the target using a control interface in order to complete the trial. After a 2-sec dwell time, the first trial was completed. Then, a blue target was shown at the home position 2 at the top of the screen and the subject had to steer the cursor into the home position 2. When the word “GO” was displayed on the screen, the subject had to steer the cursor again into the target and complete the extension trial with a 2-sec dwell time. Finally, the blue home position 1 appeared and the subject had to steer the cursor into home position 1.

Each target reached from home position 1 or home position 2 was considered as one trial. Trunk flexion describes the movement of the trunk whereby the angle between the upright position of the trunk and the legs decreases. Contrarily, trunk extension increases the angle. The movement from home position 1 to target was considered as trunk flexion and the movement from home position 2 to target was considered as trunk extension. Each block consisted of 18 trials (9 flexion and 9 extension). Before the start of the trials, the subjects were given some time to familiarise themselves with the experiment.

The sizes of the cursor and target were 30 and 100 pixels, respectively. The dis- tances between the home position and the target area were 395, 791 and 1583 pixels. At the beginning of the experiment, the subjects were asked to sub-maximally contract the muscles where the (E) sensors were placed for 2 seconds. Instructions were given that it should not cause any discomfort. This also applied to the force measurements, whereby the subjects were asked to flex or extend their trunk, or to push / pull their feet sub-maximally. The sub-maximum values were used to nor- malise the control signal. The (E) sensors were placed on the gastrocnemius/tibialis muscles of two subjects and on the quadriceps of the third subject due to spikes on the (E) signals coming from the gastrocnemius. The same researchers placed the (E) sensors on all the cases following the SENIAM guidelines [15].

In the case of the active experiment, the subjects used the Trunk Drive assistive device in order to perform the experiment [11]. No trunk support setup was used for the static experiment.

#### Parameter tuning

During the active experiment, the researchers tuned the virtual mass and damping values of the admittance model for every subject and for every control interface. If the cursor was moving too slow, the virtual mass was decreased until the subject felt comfortable with the current speed. To avoid sudden movements with a high frequency, the virtual damping was increased. The virtual mass and virtual damping made the active trunk support appear as a physical system, which is common for physical human robot interaction [16]. On the other hand, since there was no phys- ical system in the static experiment, we chose to use a different control approach. Here, the percentage of the sub-maximum contraction, together with a saturation and the dead zone, were tuned by the researchers for each subject individually to achieve the best performance. A decrease in the percentage of the sub-maximum contraction led to faster movements, whereas an increase led to slower and more fatiguing movements. The saturation ensured that a certain speed value could not be exceeded, so the speed remained bounded. Finally, the dead zone ensured that only the voluntary signals would move the cursor.

#### Experimental duration

The number of the control interfaces was different for the active and the static experiment and is described below.

The subjects started the active experiment in the following order: (J), (FS), (E), (FF), consisting of 3 blocks of 18 trials resulting in 54 trials in total. We kept the number of trials per control interface close to the number of trials of a previous study with people with DMD [7].

The subjects started the static experiment with (FF) and then (E) consisting of 1 block of 18 trials. These two control interfaces were considered the most feasible ones since one hand was required to perform the tasks and would not have been available to operate the active trunk support assistive device with the (J).

### Signal acquisition

The sensor signals were processed using a computer (xPC target, MathWorks Inc., USA) and a data acquisition card (PCI-6229; National Instrument Corp., USA). The sampling frequency was set at 1kHz with a 16-bit resolution.

### Signal processing

All the average signal amplitudes, with a rest state of 2 seconds, were subtracted and the signals were divided by a sub-maximum contraction value. Envelope de- tection was applied afterwards to the (E) signals using a high-pass Butterworth (40Hz), full wave rectifier and a low-pass Butterworth at (2Hz). No filtering was used for the (J), (FS) sensor and (FF). Regarding (FS), gravity compensation was obtained and subtracted from the voluntary force. Gravity compensation was ob- tained during slow trunk flexion (0.05 rad/sec) where the subjects had to fully relax their trunk. The muscle activation signals were measured with wireless (E) sensors (Trigno Delsys, USA) and the force with a six DoF load sell (ATI mini45).

## Additional file

**Additional file 1.** Experimental file data.

## Data Availability

All data is contained within the manuscript and Additional file 1.

## Abbreviations

DMD: Duchenne muscular dystrophy
HRQoL: surface electromyography Health Related Quality of Life
